# Doppler Ultrasound Assessment of Blood Flow Indices in Childbearing Age Women Across the Menstrual Cycle

**DOI:** 10.3390/medicina61030389

**Published:** 2025-02-24

**Authors:** Iman Akef Khowailed, Lena Volland, Ibrahim Moustafa, Cheryl Peters-Brinkerhoff, Abdulrahman M. Alsubiheen, Haneul Lee

**Affiliations:** 1 Physical Therapy Program, College of Health Sciences, University of Sharjah, Sharjah 27272, United Arab Emirates; iabuamr@sharjah.ac.ae; 2 National Beeding Disorders Foundation, New York, NY 10020, USA; 3 Doctor of Physical Therapy Program, Springfield College, Springfield, MA 01109, USA; cpeters-brinkerhoff@springfield.edu; 4 Department of Health Rehabilitation Sciences, College of Applied Medical Sciences, King Saud University, Riyadh 11433, Saudi Arabia; aalsubiheen@ksu.edu.sa; 5 Department of Physical Therapy, College of Medical Science, Gachon University, Incheon 21936, Republic of Korea

**Keywords:** menstrual cycle, blood flow, oral contraceptive, peripheral vascular function, hormonal fluctuations, Doppler ultrasound

## Abstract

*Background and Objectives:* This study investigates the effects of hormonal fluctuations during the menstrual cycle and oral contraceptive (OCP) cycles on peripheral vascular circulation in the lower limbs of healthy childbearing-age women across different phases of the menstrual cycle. *Materials and Methods:* Fourteen eumenorrheic non-oral contraceptive (non-OCP) users (mean age 28.9 ± 3.5 years; height 165.0 ± 5.8 cm; weight 66.8 ± 11.2 kg) were evaluated during the follicular and ovulatory phases. Fifteen monophasic oral contraceptive (OCP) users (mean age 26.4 ± 2.67 years; height 162.3 ± 8.1 cm; weight 62.0 ± 9.8 kg) were assessed during their placebo and active pill phases. Doppler recordings of the femoral and popliteal arteries were obtained, and standard Doppler indices (systolic/diastolic ratio, pulsatility index, and resistance index) were analyzed across the menstrual and OCP cycles. *Results:* There were no significant interactions in the standard Doppler indices for the popliteal and femoral arteries between the menstrual phases and user groups (*p* > 0.05). Additionally, no significant group effects were observed between non-OCP users and OCP users, nor were there significant phase effects in any of the Doppler index variables (*p* > 0.05). *Conclusions:* Peripheral vascular function remained stable across menstrual and OCP phases, suggesting minimal impact of hormonal fluctuations on blood flow characteristics in young, healthy females.

## 1. Introduction

Vascular function is dynamically regulated by complex physiological mechanisms, with endogenous hormonal fluctuations playing a critical role in hemodynamic stability [1,2]. Among these hormones, 17β-estradiol is widely recognized for its vasodilatory properties, primarily mediated through endothelial nitric oxide (NO) signaling, which contributes to vascular tone regulation and blood flow dynamics [3,4,5]. Throughout the menstrual cycle, endogenous estrogen concentrations fluctuate, potentially influencing peripheral vascular function. Similarly, in individuals using oral contraceptives (OCPs), exogenous hormones may modulate vascular responses. However, despite extensive research on the systemic effects of estrogen, its impact on lower limb vascular function across different menstrual cycle phases and under exogenous hormonal influence remains inadequately characterized. Given the widespread use of hormonal contraceptives and their potential effects on vascular physiology, it is essential to determine whether exogenous hormonal modulation alters vascular function [6].

Existing literature suggests that fluctuations in estradiol across the menstrual cycle may influence vascular responses, with potential implications for endothelial health and cardiovascular function [2]. However, conflicting findings complicate the interpretation of these effects, as some studies report phase-dependent alterations in vascular function, while others suggest minimal or no significant variations [2]. The study by Williams et al. [2] assessed vascular function using carotid artery stiffness, pulse wave velocity (PWV), flow-mediated dilation (FMD), and nitroglycerine-mediated dilation (NMD). Their findings indicated minimal influence of menstrual and oral contraceptive pill cycles on vascular outcomes, highlighting the complexity of hormonal effects on vascular physiology [2].

17β-estradiol, the primary and most potent form of estrogen, plays a key role in regulating reproductive and vascular functions. In women of childbearing age, its levels fluctuate across the natural menstrual cycle and OCP cycle, with notable surges during the follicular and ovulatory phases, which are implicated in modulating vascular tone and endothelial function [3,4,5]. Estradiol’s vasodilatory effects are mediated through the activation of endothelial NO synthase (eNOS), leading to increased NO production, reduced vascular resistance, and improved blood flow regulation, particularly in the context of flow-mediated vasodilatation [7].

While extensive research has been conducted on estradiol’s vascular effects, inconsistencies remain regarding its influence on peripheral circulation, particularly in the lower limbs [2]. Some studies suggest estradiol enhances endothelial function, improving vasodilation and arterial compliance, while others indicate that its influence on vascular resistance may be minimal or dependent on additional factors, such as progesterone interactions and autonomic regulation [6,7].

Doppler ultrasound has emerged as a valuable tool for non-invasive vascular assessment, offering real-time measurements of hemodynamic parameters, including pulsatility index (PI), resistance index (RI), and systolic-to-diastolic ratio [8]. Unlike (FMD), which requires an external stimulus to assess endothelial function, Doppler ultrasound enables continuous monitoring of vascular changes under physiological conditions [1]. Although Doppler ultrasound has been extensively applied in obstetric and cerebrovascular studies, its potential for evaluating menstrual cycle-related vascular changes in the lower limbs remains underexplored [2]. Prior research has demonstrated its sensitivity to hemodynamic variations in response to hormonal fluctuations, particularly in cerebral circulation [8]. In a study assessing changes in cerebral blood flow across different menstrual cycle phases, Brackley et al. (1999) found significant differences in PI and RI in the middle cerebral artery between the follicular and luteal phases, highlighting the ability of Doppler ultrasound to detect physiological vascular changes [8].

Despite extensive research on systemic vascular changes, the influence of hormonal fluctuations on lower limb hemodynamics across distinct phases of the menstrual and OCP cycles remains inadequately characterized. This study investigates cycle-dependent variations in blood flow indices, offering new insights into the vascular regulatory mechanisms underlying hormonal modulation in premenopausal women.

## 2. Materials and Methods

### 2.1. Ethical Approval

All study procedures and protocols received approval from the Institutional Review Board at the university. Written informed consent was obtained from all participants before their involvement in the study.

### 2.2. Participants

A total of thirty-four healthy, normotensive, nondiabetic, nonsmoking women with regular menstrual cycles were recruited from the university campus. This group included 17 childbearing-age, eumenorrheic women who did not use oral contraceptives (non-OCP) and 17 women who were regular users of monophasic OCP for over six months. All participants were nonsmokers, had a body mass index (BMI) within a normal range, and had no history of cardiovascular, metabolic, or endocrine disorders. Individuals were excluded if they were taking any medications other than oral contraceptives, including antihypertensives, lipid-lowering drugs, or other vasoactive agents. Additional exclusion criteria included a history of venous thromboembolism, hypertension, diabetes, thyroid dysfunction, irregular menstrual cycles (for non-OCP users), pregnancy, lactation, excessive alcohol consumption, or engagement in extreme exercise regimens that could influence vascular function. Non-OCP participants were on a cyclic, low-dose monophasic OCP pill containing a consistent amount of synthetic estradiol and progesterone in each active pill (between 20 and 35 µg of ethinyl estradiol and between 100 and 200 µg of progestin) for a period of 21 days, followed by 7 days of inactive sugar pills. Non-OCP participants were excluded if they had irregular menstrual cycles (defined as an average cycle length of less than 21 days or more than 35 days), were pregnant, or had used contraceptive pills in the previous year. OCP participants were excluded if they had used progestin-only pills, patches, rings, or intrauterine devices.

The sample size was calculated using G Power 3.1.9.4 (Heinrich Heine University Düsseldorf, Düsseldorf, Germany) [9]. A moderate effect size (Cohen’s *f*) of 0.25 was utilized for repeated measures with a within–between interaction [10], along with an α error probability of 0.05 and a power of 0.80. Based on these parameters, a total of 34 participants were determined to be necessary to achieve clinical significance.

### 2.3. Procedures

For the non-OCP participants, the menstrual cycle was monitored for two months prior to the initiation of testing procedures. To confirm the ovulatory phase, participants were provided with an ovulation predictor kit for home use, with instructions based on their menstrual history. They used the Clearblue Advanced Digital Ovulation Test (Swiss Precision Diagnostics GmbH, Geneva, Switzerland), which measures the varying levels of luteinizing hormone (LH) and estrone-3-glucuronide concentrations [11]. Ovulation typically occurs within 24–36 h following an LH surge [12]. For instance, in a subject with a standard 28-day cycle, with day 1 marking the start of menstruation, non-OCP participants began using the ovulation kit on day 7, continuing daily with their first-morning urine sample until ovulation was detected between days 12 and 14. The test was performed by either holding a test stick in the urine stream for five seconds or collecting urine in a paper cup and immersing the test stick in it for 20 s. When seeing a positive result, participants were instructed to contact the primary investigator to schedule data collection within the following 48 h.

For the OCP participants, the testing during the follicular phase occurred during the inactive pill phase (days 1–3, using sugar pills or no pills), while testing for the ovulatory phase took place during the active pill phase (days 12–14), with the first sugar pill considered day 1. Data collection during the placebo phase occurred at least 24 h after the last active pill to ensure minimal residual hormone levels.

All tests were conducted in a controlled environment at a consistent time of day (within ±30 min). Participants were instructed to avoid caffeine and alcohol for at least 12 h before testing and to consume the same meal three hours beforehand. They were also asked to maintain consistent physical activity levels, refraining from moderate-to-vigorous exercise for 24 h before each visit.

### 2.4. Measurements

#### 2.4.1. Power Doppler

Power Doppler (PD) signal assessment was performed using the musculoskeletal setting of the Next Generation LOGIQ e ultrasound machine (General Electric, Fairfield, CT, USA). Participants were instructed to lie supine to evaluate the femoral artery and in a prone position to assess the popliteal artery. PD signals were analyzed within the soft tissue components of the knee joint, including muscle fibers, tendons, and fat pads. The PD signals were rated according to a previously validated and widely accepted scoring algorithm, which assigns a score ranging from 0 (indicating no signal) to 3 (indicating confluent vessels in more than 50% of the target tissue) [13].

#### 2.4.2. Color Doppler

**Figure 1:**
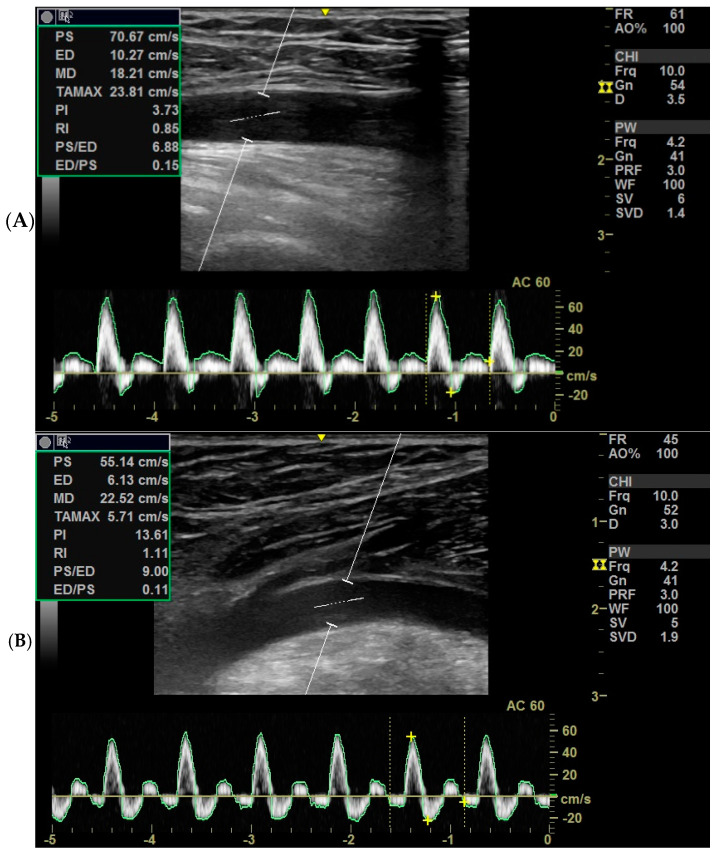
Standard Doppler indices of (**A**) the femoral artery and (**B**) the popliteal artery.

**Figure 2:**
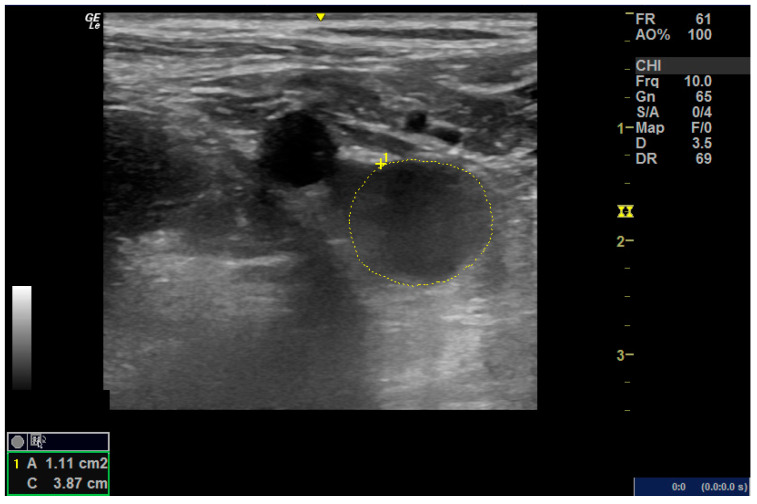
Cross-sectional area of the femoral artery.

Color Doppler techniques were conducted using the vascular setting of the same ultrasound machine (Next Generation LOGIQ e ultrasound; General Electric, Fairfield, CT, USA). Participants were positioned supine while obtaining short- and long-axis images of the femoral artery in the anterior groin region. For the popliteal artery, participants assumed a prone position with their knees comfortably extended, and the transducer was placed in the popliteal fossa to capture the necessary images. Grayscale pulsed and color Doppler flow imaging were employed during both systolic and diastolic intervals. Cross-sectional areas of the femoral and popliteal arteries were calculated using lumen measurements from transverse images, as per the GE measurement settings (Figure 1A,B). The resistance index, systolic/diastolic ratios, and pulsatility index were computed to evaluate blood flow from the generated waveform [14] (Figure 2).

Doppler ultrasound was performed to assess the popliteal and femoral arteries, recording several hemodynamic parameters. Peak Systolic Velocity (PS, cm/s) represents the maximum blood flow velocity observed during the systolic phase. End-Diastolic Velocity (ED, cm/s) refers to the blood flow velocity at the end of diastole, with cases where ED = 0 excluded from averaging to prevent skewed results. Minimum Absolute Velocity in the Diastolic Cycle (MD, cm/s) represents the lowest recorded velocity during diastole. Time-Averaged Maximum Velocity (TAMAX, cm/s) is the maximum velocity averaged across multiple cardiac cycles, assessing peak flow dynamics. Cross-Sectional Area (CS, mm^2^) was estimated based on arterial diameter using the equation CS = π × (MD/2)^2^, providing an estimate of vessel luminal area and reflecting potential structural or functional changes [15]. Each parameter was measured three times, with the average used for analysis to ensure reliability [15].

The following Doppler indices were derived from the measured parameters. Pulsatility Index (PI) was calculated as PI = (PS − ED)/MD, reflecting blood flow pulsatility and arterial compliance. Resistive Index (RI) was computed as RI = (PS − ED)/PS to assess downstream vascular resistance. Systolic/Diastolic Ratio (PS/ED) was determined using PS/ED = PS/ED, evaluating arterial resistance and compliance. Diastolic/Systolic Ratio (ED/PS) was calculated as ED/PS = ED/PS, serving as an inverse measure of arterial compliance and providing insights into diastolic blood flow relative to systolic peak velocity. These indices were analyzed to assess vascular health and function, with values interpreted in the context of normal hemodynamic responses [15].

### 2.5. Statistical Analysis

Data analysis was performed using SPSS 26.0 software for Windows (IBM Corp., Armonk, NY, USA). Descriptive statistics were presented as means ± standard deviations (SD). The Shapiro–Wilk test was employed to assess the normality of continuous variables. Repeated measures ANOVA was conducted to evaluate potential interactions among the outcome variables across the menstrual phases (follicular vs. ovulatory) and the two groups (OCP users vs. non-OCP users). To account for multiple comparisons, Bonferroni correction was applied, adjusting the significance level to α = 0.0056. Additionally, multiple linear regression analysis was conducted to evaluate the potential influence of confounding variables, such as age and BMI, on vascular parameters (PI, RI, PS, ED, MD, and TAMAX).

## 3. Results

Fourteen non-OCP and 15 OCP users completed the study. Three non-OCP users and two OCP users withdrew because of scheduling conflicts, and one non-OCP user dropped out because of menstrual irregularities. The general characteristics of the participants are shown in Table 1. The non-OCP users had an average age of 28.9 ± 3.5 years, a height of 165.0 ± 5.8 cm, and a weight of 66.8 ± 11.2 kg. The OCP users had an average age of 26.4 ± 2.67 years, a height of 162.3 ± 8.1 cm, and a weight of 62.0 ± 9.8 kg. There were no significant differences in general characteristics between the two groups.

Multiple linear regression analysis was conducted to evaluate the potential influence of confounding variables, such as age and BMI, on vascular parameters (PI, RI, PS, ED, MD, and TAMAX). After adjusting for these confounders, the group variable did not show a significant association with the vascular parameters (*p*-value > 0.05) for both popliteal and femoral artery measurements. Specifically, for parameters like PI, RI, PS, ED, MD, and TAMAX, no significant differences were observed between the groups (non-OCP vs. OCP users).

Table 2 shows the vascular functions of non-OCP and OCP users during the follicular phase and ovulation. After applying Bonferroni correction (α = 0.0056), most endothelial function parameters showed no significant differences. Specifically, no significant interactions between the menstrual phase and group for any of the endothelial functions measured in the popliteal or femoral arteries. Similarly, there were no significant differences between non-OCP users and OCP users in any of the vascular function parameters, including PI, RI, PS, ED, MD, and TAMAX. However, for the popliteal artery, CS remained statistically significant (*p* < 0.0056). In contrast, no significant differences were observed for any parameters in the femoral artery.

Furthermore, no significant phase effects were detected within each group when comparing the follicular and ovulation phases.

A post-hoc power analysis indicated that the study was underpowered to detect small-to-moderate effects for most vascular parameters, requiring larger sample sizes per group to achieve 80% statistical power.

## 4. Discussion

This study adds to existing research by analyzing standard Doppler indices of peripheral vascular function in relation to the phases of the natural menstrual cycle compared to those of a monophasic OCP cycle. Unlike prior studies that predominantly focused on upper limb vasculature, this study examined the femoral and popliteal arteries, providing insights into lower limb blood flow characteristics. The key findings indicate that the standard Doppler indices for both the popliteal and femoral arteries showed no significant variation across the menstrual and OCP cycles, nor were there notable differences between the two groups. These findings suggest that hormonal fluctuations, including those induced by OCP, do not significantly impact vascular function in healthy young women.

The influence of the menstrual cycle on vascular function remains unclear, as studies present both supportive and contradictory findings [16,17,18]. Recent debates among researchers regarding the necessity of controlling for menstrual cycle phases in vascular studies [6,19] highlight the importance of more nuanced investigations into vascular responses during different phases. Our findings, which indicate stable vascular function, support the notion that peripheral vascular assessments may not require strict phase-specific control in healthy childbearing women.

Recent research challenges the historical exclusion of females in cardiovascular studies due to concerns about hormonal cycle influences. Williams et al. (2024) conducted a comprehensive investigation into the effects of natural menstrual and oral contraceptive pill cycles on vascular function and cellular regulation [2]. Their study found that while brachial artery (BA) endothelial function showed a small phase-dependent effect, this was largely explained by baseline artery diameter differences. No significant effects were observed on superficial femoral artery (SFA) endothelial function, smooth muscle function, arterial stiffness, or key cellular mechanisms, including endothelial nitric oxide synthase (eNOS) and estrogen receptor-α (ERα) protein content [2]. In agreement with these findings, our results suggest that menstrual cycle and OCP phases have a minimal impact on vascular health, reinforcing the importance of including female participants in future research without concerns about hormonal variability.

Ciccone et al. (2013) investigated the acute hemodynamic effects of intranasal 17-β-estradiol on cerebral and lower limb arterial circulation in healthy postmenopausal women [20]. Their findings demonstrated a significant increase in cerebral perfusion, as reflected by changes in the internal carotid artery’s velocity/time integral. However, no substantial alterations were observed in the posterior tibial artery, suggesting a limited effect on peripheral circulation [20]. These results align with our study, which also found minimal impact of hormonal fluctuations on peripheral vascular function in young, healthy females. The consistency between these findings reinforces the notion that estrogen’s vasoregulatory effects may be more pronounced in the cerebral circulation, while its influence on peripheral blood flow remains limited [3,4]. This highlights the complexity of estrogen-mediated vascular modulation and underscores the need for further research to elucidate the mechanisms driving these differential effects.

Ciccone et al. (2012) investigated the effects of intranasal estradiol on ophthalmic artery vasodilation in postmenopausal women [21]. Their study found significant vasodilation in the ophthalmic artery following estradiol administration, indicating that estrogen may have a more pronounced effect on specific vascular regions, particularly in the cerebral and ocular circulations [21]. This finding supports the hypothesis that estrogen’s vascular effects may be site-specific, exerting a stronger influence on certain regions, such as the brain and eyes, while having a limited effect on peripheral circulation, particularly in the lower limbs [21].

This observation aligns with the results of our study, which also found a minimal impact of hormonal fluctuations on peripheral vascular function in young, healthy females. The consistency between these findings reinforces the notion that estrogen’s vasoregulatory effects are more substantial in cerebral and ocular circulation, while peripheral blood flow remains largely unaffected by hormonal changes. By demonstrating that hormonal fluctuations, whether due to natural menstrual cycles or OCP use, do not significantly impact peripheral circulation, our findings complement those of Ciccone et al. (2012) [21], reinforcing the hypothesis that estrogen’s vascular effects are more prominent in certain vascular territories [3,4,5].

By demonstrating that hormonal fluctuations, whether due to natural menstrual cycles or OCP use, do not significantly impact peripheral circulation, our findings further support the hypothesis that estrogen’s vascular effects may be site-specific. While estrogen has been shown to enhance cerebral blood flow, its role in peripheral vascular regulation appears to be less pronounced [5]. Future studies should explore potential mechanisms underlying these differences, including receptor distribution, vascular tone regulation, and interactions with other hormonal and metabolic factors. Understanding these nuances is essential for evaluating estrogen’s broader implications for vascular health across different physiological states and age groups.

In contrast to our findings, which indicate that peripheral vascular function remains stable across menstrual and oral contraceptive phases, Ciccone et al. (2013) demonstrated that modulation of estrogen receptors can influence vascular responses during isometric muscular stress [22]. Specifically, their study found that the administration of raloxifene, a selective estrogen receptor modulator, reduced the vascular effects of isometric muscle contraction by modulating the vasomotor tone of peripheral vessels in relation to exercise [22]. This discrepancy suggests that hormonal influences on vascular function may be more pronounced under specific physiological conditions, such as isometric exercise, which were not examined in our study. Therefore, while our results indicate minimal impact of hormonal fluctuations on blood flow characteristics at rest, it is important to consider that different assessment methods and physiological conditions may yield varying outcomes [5].

Our findings support recent research showing no variations in vascular function throughout the menstrual cycle, including during the ovulatory phase when estrogen peaks and progesterone is lower [17,23,24,25]. In contrast, some studies have reported enhanced vascular function during the early and mid-luteal phases compared to the early follicular phase [26,27]. These discrepancies may be differences in vascular beds, as macrovascular and microvascular functions can react differently to hormonal fluctuations. Additionally, our findings align with those of Williams et al. (2024) [2], who found no effect of the menstrual cycle on vascular function. A study by Jekell et al. (2019) [28] also found weak correlations between macrovascular and microvascular endothelial function in individuals with hypertension, a finding echoed in rheumatoid arthritis patients [29]. Such variations suggest distinct regulatory mechanisms across different vascular beds [29,30].

Some studies have employed (FMD) to assess endothelial function, considered a gold standard for evaluating endothelial-dependent vasodilation [2,4]. In contrast, our study used Doppler ultrasonography, which measures peripheral vascular resistance and blood flow velocity. While Doppler ultrasonography does not directly assess endothelial function, it is useful for evaluating macrovascular function. These methodological differences—particularly between FMD and Doppler ultrasonography—could help explain the mixed findings observed in the literature regarding the menstrual cycle effects on vascular function [31]. Moreover, studies that utilized Doppler ultrasonography have generally focused on macrovascular structures, which may respond differently to hormonal changes compared to microvasculature [32]. These differences in methods could contribute to the varying results observed. While FMD studies report varying effects of hormonal fluctuations on endothelial function [33], our Doppler ultrasonography results suggest that these fluctuations have minimal impact on peripheral vascular health. Future studies could consider incorporating both methods to offer a more comprehensive assessment of vascular function [31].

The validity of Doppler ultrasonography for assessing vascular function is well-supported in the literature. Brackley et al. (1999) demonstrated its effectiveness in detecting hemodynamic changes across different phases of the menstrual cycle, reinforcing its role in vascular assessment [8]. A systematic review by Williams et al. (2020) also confirmed that hormonal fluctuations influence vascular responses, further supporting the use of Doppler ultrasonography in evaluating peripheral vascular function [33]. Furthermore, Brackley et al. (1999) provide robust evidence for Doppler ultrasound as a reliable tool for measuring vascular function [8]. Their research showed that Doppler-derived indices, such as (PI) and (RI), are sensitive markers of hemodynamic variations, particularly in response to hormonal fluctuations. This highlights the effectiveness of Doppler ultrasonography in detecting physiological changes in vascular function, making it a valuable method for comprehensive vascular assessment.

Despite the global prevalence of oral contraceptives [34], research on their impact on vascular function remains limited. Our findings indicate no significant effects of the active and inactive phases of the monophasic OCP cycle on vascular function, supporting previous studies that reported similar results [18]. This suggests that monophasic OCP use may provide hormonal stability without adversely affecting peripheral vascular health in active young women, though more research is needed to explore this relationship comprehensively, particularly in women with varying activity levels or cardiovascular risk factors.

This study has several limitations. The relatively small sample size may have influenced the findings. While an initial power calculation suggested 34 participants would be sufficient for detecting moderate effects, a post-hoc analysis indicated that a larger sample would be needed to detect small-to-moderate effects under the Bonferroni correction. Despite this, the sample size was adequate to detect large effects, suggesting that the lack of significant findings reflects minimal physiological differences rather than insufficient power. Another limitation is the use of Doppler ultrasonography to assess endothelial function. Although effective for evaluating blood flow indices in the femoral and popliteal arteries, the absence of flow-mediated dilatation to assess endothelial function limits the comparison to previous studies. Future studies should incorporate both methods to provide a more comprehensive assessment.

Despite these limitations, this study provides valuable insights into vascular function across menstrual and OCP phases in childbearing-age women. The findings suggest that menstrual and OCP cycle phases may not significantly influence lower limb blood flow in healthy childbearing-age women. However, larger studies incorporating additional hormonal assessments and endothelial function measurements are needed to confirm these results and further explore the relationship between hormonal fluctuations and vascular health.

## 5. Conclusions

This study found that lower limb vascular blood flow remained stable during both the early follicular and ovulatory phases of the menstrual cycle, as well as between the inactive and active phases of the OCP cycle in young, healthy women. No significant differences in vascular function were observed across these phases, indicating that the menstrual and OCP cycle phases may not significantly impact vascular blood flow in this population. These findings suggest that menstrual and OCP cycle phases may not need to be factored into assessments of vascular health in young, active women. However, further research, including larger studies with broader hormonal assessments, is essential to fully understand the relationship between hormonal fluctuations and vascular health and to determine its clinical implications for women’s cardiovascular well-being.

## Figures and Tables

**Table 1:** General participant characteristics (N = 29).

	Non-OCP Users (*n* = 14)	OCP Users (*n* = 15)
Age (years)	28.9 ± 3.5	26.4 ± 2.67
Height (cm)	165.0 ± 5.8	162.3 ± 8.1
Weight (kg)	66.8 ± 11.2	62.0 ± 9.8
Body mass index (kg/m^2^)	24.5 ± 3.8	23.5 ± 2.4

**Table 2:** Endothelial functions observed in non-OCP users and OCP users during follicular and ovulation phase.

		Non-OCP Users (*n* = 14)		OCP Users (*n* = 15)		*F*(p) ^†^	Partial η^2^
		Follicular	Ovulation	Follicular	Ovulation		
Popliteal artery	PI	21.1 ± 13.4	17.63 ± 11.72	16.7 ± 13.3	16.01 ± 15.10	0.544(0.47)	0.020
	RI	1.08 ± 0.06	1.07 ± 0.09	1.07 ± 0.13	1.07 ± 0.08	0.087(0.62)	0.003
	PS/ED	7.03 ± 3.70	7.66 ± 5.28	5.65 ± 4.47	6.44 ± 3.91	1.931(0.19)	0.067
	ED/PS	0.07 ± 0.06	0.07 ± 0.09	0.09 ± 0.12	0.09 ± 0.10	0.153(0.70)	0.006
	PS	58.19 ± 10.67	55.55 ± 14.62	55.47 ± 14.22	58.10 ± 8.93	0.647(0.43)	0.023
	ED	4.79 ± 3.53	3.63 ± 5.04	4.68 ± 5.42	3.39 ± 4.24	0.005(0.94)	0.000
	MD	22.84 ± 4.69	22.13 ± 5.33	19.81 ± 5.77	19.71 ± 5.99	0.004(0.96)	0.000
	TAMAX	5.77 ± 4.70	6.65 ± 3.19	8.15 ± 6.46	7.58 ± 3.45	0.178(0.68)	0.007
	CS	0.23 ± 0.09	0.34 ± 0.24	0.33 ± 0.17	0.29 ± 0.08	3.920(0.06)	0.127
Femoral artery	PI	5.33 ± 1.83	9.29 ± 9.69	7.48 ± 6.09	9.82 ± 14.81	0.086(0.77)	0.003
	RI	0.93 ± 0.07	0.94 ± 0.11	0.94 ± 0.08	0.95 ± 0.09	0.083(0.78)	0.003
	PS/ED	6.66 ± 3.09	5.16 ± 3.13	4.81 ± 3.00	7.25 ± 4.95	3.804(0.07)	0.123
	ED/PS	0.07 ± 0.06	0.08 ± 0.09	0.09 ± 0.12	0.08 ± 0.09	0.337(0.57)	0.012
	PS	74.35 ± 3.93	68.66 ± 14.02	75.78 ± 2.34	74.30 ± 5.73	0.024(0.88)	0.001
	ED	5.20 ± 4.49	5.32 ± 6.10	4.61 ± 4.74	5.94 ± 6.66	0.064(0.80)	0.002
	MD	26.98 ± 5.45	27.58 ± 4.92	30.16 ± 3.53	27.86 ± 5.72	2.090(0.16)	0.072
	TAMAX	18.30 ± 7.53	17.59 ± 9.63	17.75 ± 8.48	17.97 ± 8.39	0.190(0.67)	0.007
	CS	0.54 ± 0.18	0.68 ± 0.21	0.66 ± 0.28	0.64 ± 0.20	1.460(0.24)	0.051

Abbreviations: PI, pulsatility index; RI, resistance index; PS, peak systolic velocity; ED, end-diastolic velocity; MD, minimum absolute velocity in diastolic cycle; TAMAX, time-averaged maximum velocity obtained from vascular trace measurement; CS, cross-sectional area of the artery. † Interaction between menstrual phase and group.

## Data Availability

The datasets generated during this study are available from the corresponding author upon reasonable request.
